# Post-column coulometric generation and cyclic voltammetric identification of the free radical of the antineoplastic agent etoposide (VP 16-213)

**DOI:** 10.1155/S1463924688000252

**Published:** 1988

**Authors:** H. H. J. L. Ploegmakers, W. J. van Oort

**Affiliations:** Department of Analytical Pharmacy Faculty of Pharmacy State University of Utrecht Catharijnesingel 60 Utrecht 3511 GH The Netherlands

## Abstract

It has been suggested that etoposide can be transformed ‘in vivo’ into a radical intermediate, which may be involved in the irreversible binding of etoposide to microsomal proteins and in DNA damage. To investigate some physico-chemical properties, the on-line coulometric production of this free radical and its subsequent cyclic voltammetric detection is described. For the synthesis, a coulometric ESA Coulochem 5100A module, equipped with an ESA 5010 analytical cell, has been used. For its detection the computerized cyclic voltammetric detection system after HPLC controlled by an Apple IIe computer, using home-made software and a home-made glassy-carbon wall-jet detector, has been used. Application of a ramdisk allows storage of 68 cyclic voltammograms. In a methanol-phosphate buffer (pH=8) (40:60 w/w) containing mobile phase, the forward scan of the on-line cyclic voltammogram of etoposide shows two oxidation steps, caused by a double one-electron transfer. Post-column electrolysis at +500 mV versus the H^+^/H2 reference electrode results into total disappearance of the first oxidation step, whereas a reduction wave arises. The limiting current of this wave, and the remaining second oxidation wave, are of equal heights, indicating one-electron processes. Under these conditions, the half-life time of the product, and consequently the free radical, is 100 s, determined by stopped-flow analysis. Decreasing the pH of the buffer in the mobile phase to pH 2.2 results in merging of the two oxidation steps to a single two-electron transfer, disabling radical formation. Under physiological conditions (‘in vivo’) the established half-life time of the radical enables participation of the intermediate in metabolic processes.


Journal of Automatic Chemistry, Vol. 10, No. 3 (July–September 1988), pp. 135–139

### Post-column coulometric generation and cyclic voltammetric identification of the free radical of the antineoplastic agent etoposide (VP 16–213)

**H. H. J. L. Ploegmakers and W. J. van Oort**

*Department of Analytical Pharmacy, Faculty of Pharmacy, State University of Utrecht, Catharijnesingel 60, 3511 GH Utrecht, The Netherlands*

*It has been suggested that etoposide can be transformed 'in vivo' into a radical intermediate, which may be involved in the irreversible binding of etoposide to microsomal proteins and in DNA damage. To investigate some physico-chemical properties, the on-line coulometric production of this free radical and its subsequent cyclic voltammetric detection is described. For the synthesis, a coulometric ESA Coulochem 5100A module, equipped with an ESA 5010 analytical cell, has been used. For its detection the computerized cyclic voltammetric detection system after HPLC controlled by an Apple IIe computer, using home-made software and a home-made glassy-carbon wall-jet detector, has been used. Application of a ramdisk allows storage of 68 cyclic voltammograms. In a methanol-phosphate buffer (pH = 8) (40 : 60 w/w) containing mobile phase, the forward scan of the on-line cyclic voltammogram of etoposide shows two oxidation steps, caused by a double one-electron transfer. Post-column electrolysis at +500 mV versus the $H^+/H_2$ reference electrode results into total disappearance of the first oxidation step, whereas a reduction wave arises. The limiting current of this wave, and the remaining second oxidation wave, are of equal heights, indicating one-electron processes. Under these conditions, the half-life time of the product, and consequently the free radical, is 100 s, determined by stopped-flow analysis. Decreasing the pH of the buffer in the mobile phase to pH = 2·2 results in merging of the two oxidation steps to a single two-electron transfer, disabling radical formation. Under physiological conditions ('in vivo') the established half-life time of the radical enables participation of the intermediate in metabolic processes.*

### Introduction

Etoposide (VP 16–213) and teniposide (VM 26), two semi-synthetic podophyllotoxin derivatives, are promising anti-tumor agents for the treatment of small cell lung carcinoma [1 and 2], paediatric acute lymphoblastic leukemia [3] and bladder cancer [4]. Although the exact mechanism of action is unknown, Loike [5] has reported the effect of various podophyllotoxin derivatives on the cellular degradation of DNA in HeLa cells: teniposide, etoposide and podophyllotoxin reversibly inhibit the uptake of thymidine, uridine, adenosine and guanosine [5–7]. The addition of etoposide to adriamycin, cis-platinum or cyclophosphamide decreases toxicity [8]; if podophyllotoxin (not containing a phenolic hydroxyl group) is added instead of etoposide, no such activity is observed. Sinha *et al.* [9–11] suggest that lipid peroxidation, induced by, for example, adriamycin is inhibited by etoposide, related to its ability to utilize the generated hydrogen peroxide for its own activation. This peroxidative process produces the oxygen-centred free radical intermediate of etoposide.

The study of Sinha *et al.* [10] also proves that etoposide does not affect the initial formation of the anthracycline free radical nor that etoposide reacts with the superoxide anion to form a secondary radical.

In addition, preliminary reports by van Maanen *et al.* [12], Wozniak *et al.* [13] and Sinha and Myers [14] have hypothesized that etoposide forms a radical intermediate, which may be involved in the irreversible binding of etoposide to microsomal proteins and in DNA damage.

Sinha *et al.* [11] have generated the free radical of etoposide enzymatically (horseradish peroxidase/hydrogen peroxide) and van Maanen *et al.* [15] prepared it chemically (ferrous/persulphate), enzymatically (myeloperoxidase and horseradish peroxidase/hydrogen peroxide) and electrochemically (+ 550 mV versus Ag/AgCl electrode). All three methods brought about a single one-electron transfer, resulting into the formation of a relatively stable free radical, detectable by electron spin resonance (e.s.r.).

Up to now, the arise of radicals has mostly been proven by e.s.r.. Electrochemical methods like cyclic voltammetry, however, may provide more detailed information on oxidative and reductive electron transfer processes. In a past communication [16], the authors described a computerized cyclic voltammetric detection system after HPLC, enabling separation, detection and determination of electro-active compounds. The application of a chromatographic system, using an oxygen-deficient mobile phase, enables almost anaerobic conditions to be created, and the drug and oxygen, which is always present in the sample, to be separated.

At pH-values larger than 2·5, etoposide can be oxidized in two distinguishable one-electron steps [16 and 17]. Therefore, as the first oxidation step results in the formation of a stable free radical [15], it is our aim to generate the radical coulometrically at constant potential, on-line and post-column under almost anaerobic conditions, and to subsequently characterize the electrolysis product.

### Experimental

### *Hardware and software*

The computerized, on-line data acquisition and processing system used, has been previously described [16].

Post-column, the system has been coupled with an ESA Coulochem 5100A module using an ESA 5010 analytical cell, a Waters Associates model 440 u.v.-absorbance detector (280 and 365 nm) and a home-made oxidative wall-jet detector [16].

To detect oxygen on-line, a Princeton Applied Research Model 310 polarographic detector has been applied.

### *Chemicals and samples*

Etoposide was supplied by Bristol-Myers Nederland BV, Bussum, The Netherlands. The mobile phase consisted of methanol/0·020 M phosphate buffer (40:60 w/w). The composition of the buffer was varied from experiment to experiment. Exact compositions have been presented for each experiment. In order to separate etoposide and dissolved oxygen, the samples were injected on a Waters Associates μCN (P/N 84042) column. As a stock solution, 10 mg of etoposide was dissolved in 10·0 ml methanol p.a. Merck.

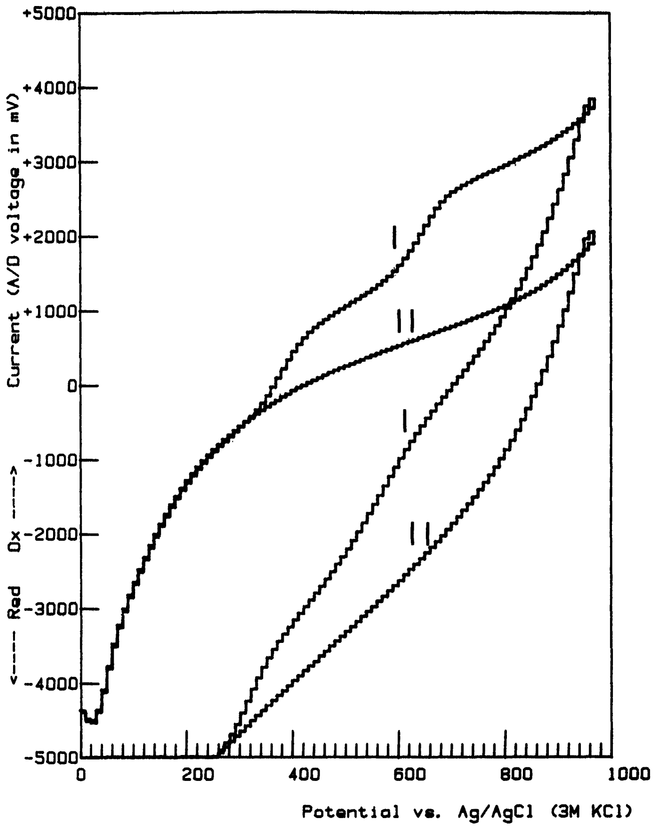

*Figure 1. On-line cyclic voltammograms of etoposide (I) and blank (II) after HPLC. Conditions: current range 0·5 μA F.S.; 30 μg injected; mobile phase, methanol/0·020 M phosphate buffer pH = 8 (40:60 w/w); Waters Associates μCN (P/N 84042) column; 1 ml/min flow rate; scan speed 200 mV/s; scan range 0 to +1000 mV versus the Ag/AgCl (3 M KCl) electrode.*

### Results and discussion

Etoposide can be oxidized electrochemically at the glassy carbon electrode (GCE). The forward scan of the batchwise cyclic voltammogram of the compound at pH 2·5–14 shows two oxidative peaks, caused by a double one-electron transfer [17]. The first electron-release is a pH-dependent reversible process, after which a neutral free radical results [15]. The second step is the formation of the cation, followed by rapid solvolytic conversion into an ortho-quinone. The irreversibility of the second electron-release is at least partly caused by the rapid conversion of the primary electrolysis product [17].

The electrochemically produced free radical of etoposide has been described by van Maanen *et al.* [15]. By coupling an electrochemical flow-through cell and an e.s.r. aqueous flat cell, the free radical was produced and detected on-line. The electrolysis at constant potential was carried out using a platinum gauze working electrode (+500 mV versus Ag/AgCl) in 1 mM Tris buffer (pH = 7·4) (0·1 M NaCl) and in 1 mM Tris buffer (pH = 7·4) (0·1 M NaCl) containing 20% methanol (v/v). Under these conditions, the half-life times of the free radical were 257 s and 361 s respectively.

In this paper, the ESA Coulochem system has been used to generate the free radical on-line after HPLC. In this kind of system, the cyclic voltammograms of etoposide show properties similar to batchwise results [16] with the exception of sigmoidal-shaped curves [18] (waves) being generated, instead of peaks (figure 1). In the HPLC system described here, the retention times of oxygen and etoposide are 6 min 00 s and 7 min 30 s.

The benefits of this coulometric system, equipped with a graphite powder working electrode instead of platinum gauze, include a high yield (theoretically a 100% conversion) and short electrolysis time, the latter being especially important in case of relatively short-lived free radicals.

In the Coulochem detector, an unconventional reference electrode has been used by the supplier, using the $H_2/H^+$ couple [19]. As the exact potential of this electrode was not given by the manufacturer, the working electrode potential of the coulometric system had to be chosen experimentally. Varying the coulometric electrolysis potential in the HPLC system from +200 mV up to +1200 mV versus the $H_2/H^+$ couple at pH = 8, no electrochemical activity was observed at +200 mV and +300 mV. At +400 mV, however, the limiting current of the first oxidation step of etoposide decreases, whereas a reduction wave arises. Changing the electrolysis-potential to +500 mV results into total absence of the first oxidation step in the forward scan and progress of the reduction wave (see figure 2[*a*]). The reverse scan of the cyclic voltammogram also shows a reduction wave (figure 2[*a*]). Decreasing the scan limit from +1000 mV to +600 mV versus the Ag/AgCl electrode, proves that the reduction waves in the forward and reverse scans are related (figure 2[*b*]).

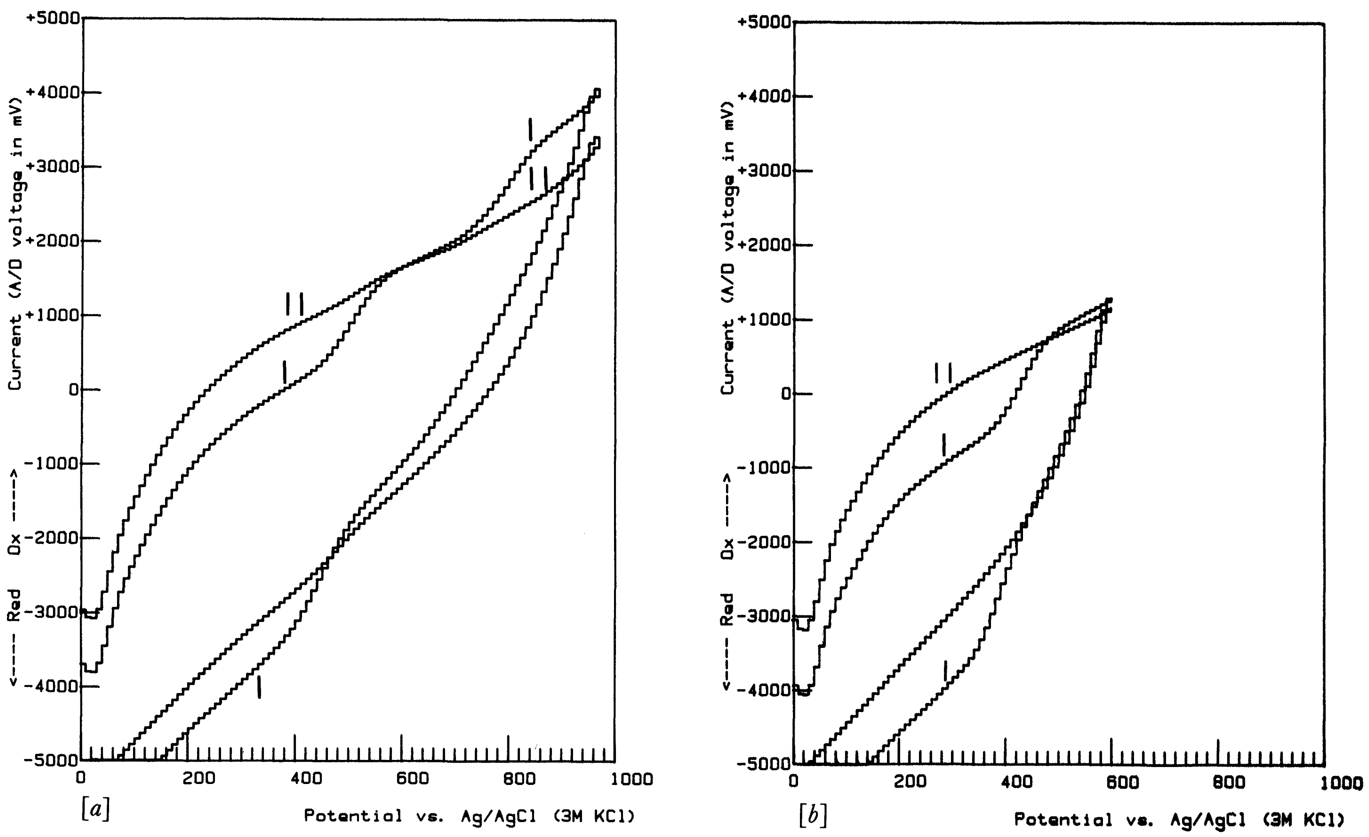

*Figure 2. On-line cyclic voltammograms of the electrolysis-product of etoposide (I) and blank (II) after HPLC. Conditions: current range 0·5 μA F.S.; 30 μg injected; mobile phase, methanol/0·020 M phosphate buffer pH = 8 (40 : 60 w/w); Waters Associates μCN (P/N 84042) column; 1 ml/min flow rate; scan speed 200 mV/s; coulometric potential +500 mV versus $H^+/H_2$ couple; [a] scan range 0 to +1000 mV versus the Ag/AgCl (3 M KCl) electrode; [b] scan range 0 to +600 mV versus the Ag/AgCl (3 M KCl) electrode.*

Increasing the electrolysis-potential to +900 mV results into the arise of a new oxidation wave and increase and cathodic shift of the reduction wave (figure 3), whereas electrolysis at +1200 mV no longer yields either oxidizable nor reductable substances. Decreasing the pH does not lead to unexpected electrochemical changes. As the first oxidation step is pH-dependent and the second pH-independent, at pH = 6 the first step has been shifted in anodic direct by 57 mV/pH [17]; at pH = 4 the two oxidation steps have almost been merged. Electrolysis at pH = 4 shows that the reduction wave still exists but its limiting current has diminished (figure 4). At pH = 2·2, the two oxidation steps of etoposide have been merged completely, whereas electrolysis (+800 mV) does not result into a reduction wave.

In pH-range 2–9·7, etoposide partly occurs in the phenolate form, which can be oxidized more easily in a similar way to other conjugate bases [17]. The first reaction-step is favoured by increasing pH; the second reaction-step is pH independent. Decreasing the pH leads to a reduction in the phenolate/phenol ratio, which results into anodic shifting of the first step; as the second step is pH-independent, the shift leads to merging of both steps.

The free radical of etoposide arises at the first oxidation step. At pH = 8, allowing two separate one-electron steps (figure 1), the radical, indicated by the disappearance of the first oxidation wave and the generation of a reduction wave (figure 2[*a*]), has been generated. The limiting currents of the remaining oxidation wave and of the reduction wave are both of equal heights, indicating one-electron processes. At pH = 4 only a low yield has been obtained (figure 4) whereas at pH = 2·2, the two oxidation steps of etoposide are merged together, disabling the formation of the radical. These facts suggest that the arise of the free radical of etoposide is conditioned by the existence of two separated oxidation steps (pH > 4).

Electrolysis at +900 mV (pH = 8) (figure 3) results in an increase and cathodic shift of the reduction wave and the generation of a reversible couple in the forward scan, probably caused by reduction of the on-line coulometrically produced orthoquinone to a catecholic compound, followed by its oxidation.

During electrolysis, there is a relationship between coulometric activity and u.v. absorbance at 365 nm (see table 1), in addition to aromatic absorption in the 280 nm range. Free radicals of 2,4,6-alkyl-substituted phenols

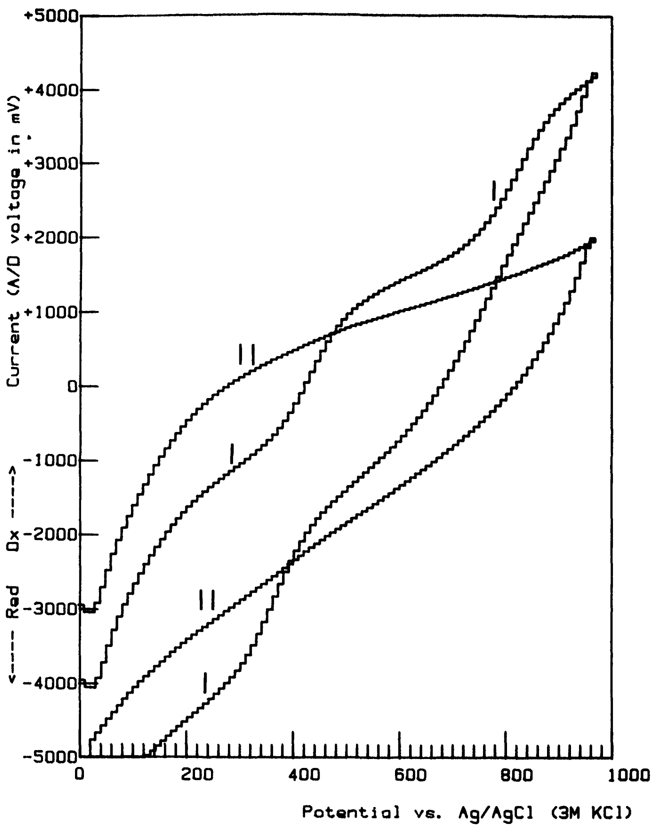

*Figure 3. On-line cyclic voltammograms of the electrolysis-product of etoposide (I) and blank (II) after HPLC. Conditions: current range 0·5 μA F.S.; 30 μg injected; mobile phase, methanol/0·020 M phosphate buffer pH = 8 (40 : 60 w/w); Waters Associates μCN (P/N 84042) column; 1 ml/min flow rate; scan speed 200 mV/s; coulometric potential +900 mV versus $H^+/H_2$ couple; scan range 0 to +1000 mV versus the Ag/AgCl (3 M KCl) electrode.*

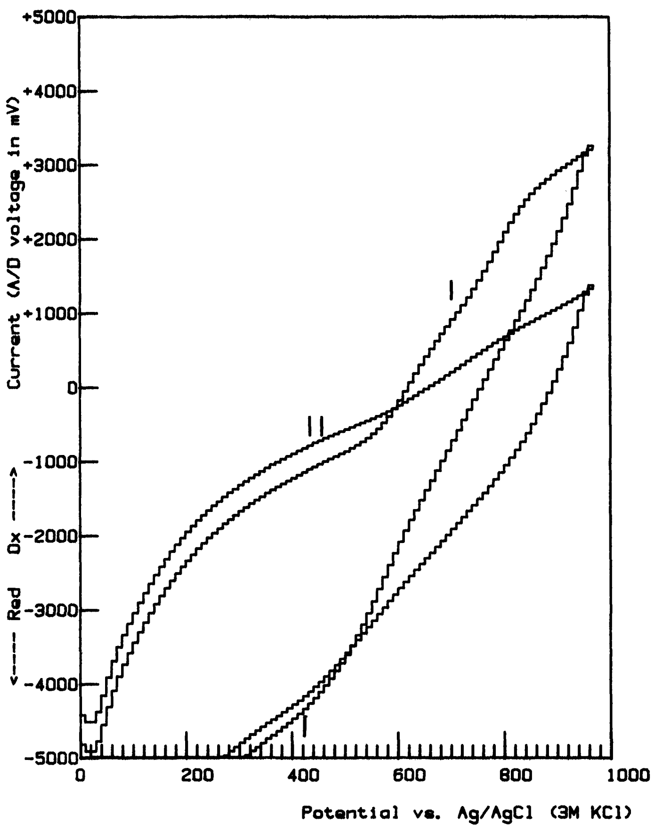

*Figure 4. On-line cyclic voltammograms of the electrolysis-product of etoposide (I) and blank (II) after HPLC. Conditions: current range 0·5 μA F.S.; 30 μg injected; mobile phase, methanol/0·020 M phosphate buffer pH = 4 (40 : 60 w/w); Waters Associates μCN (P/N 84042) column; 1 ml/min flow rate; scan speed 200 mV/s; coulometric potential +700 mV versus $H^+/H_2$ couple; scan range 0 to +1000 mV versus the Ag/AgCl (3 M KCl) electrode.*

*Table 1. The relation between coulometric potential, coulometric oxidative activity and u.v. absorbance at pH = 8.*

| Electrolysis potential Coulochem detector (mV versus $H^+/H_2$) | Oxidation in Coulochem | Absorbance 280 nm | Absorbance 365 nm |
|---|---|---|---|
| +200 | — | + | — |
| +300 | — | + | — |
| +400 | + | + | + |
| +500 | + | + | + |
| +600 | + | + | + |
| +700 | + | + | + |
| +800 | + | + | + |

may undergo disproportionation or dimerization forming quinoid compounds. Especially when substituents at the para-position are relatively small (t-butyl, isopropyl, ethyl or methyl), disproportionation may occur. This relatively slow process, which is only of importance in long preparative electrolysis, results in the formation of a phenol and a quinone-methide. Another possibility is the fast dimerization to paraquinol, which only occurs when the primary alkyl-substituents are small (n-propyl, ethyl or methyl) [20]. In the case of etoposide, however, none of these conditions can be fulfilled due to the presence of the large A, B, C and D ring system at the para-position [17].

The oxidation product of etoposide at +850 mV versus the Ag/AgCl electrode (pH = 4·6) is orthoquinone. This compound shows absorption maxima at 365 and 470 nm [17]. However, it is unlikely that coulometric electrolysis at +400 mV versus the $H^+/H_2$ couple will result into the formation of an orthoquinone.

Therefore, the arise of u.v.-absorbance at 365 nm, simultaneously with coulometric activity at +400 mV, cannot be explained by the existence of quinoid compounds but is probably caused by the free radical itself.

To determine the half-life time of the radical, the stopped-flow method was used. After injecting the compound, the cyclic voltammograms are monitored on-line on screen. As soon as the radical enters the detector-cell, the flow is stopped and the scans are stored in memory.

Figure 5 shows a number of cyclic voltammograms of the electrochemically produced etoposide free radical. It shows that the radical is converted totally to a non-oxidizable compound depending on time. Upon perform-

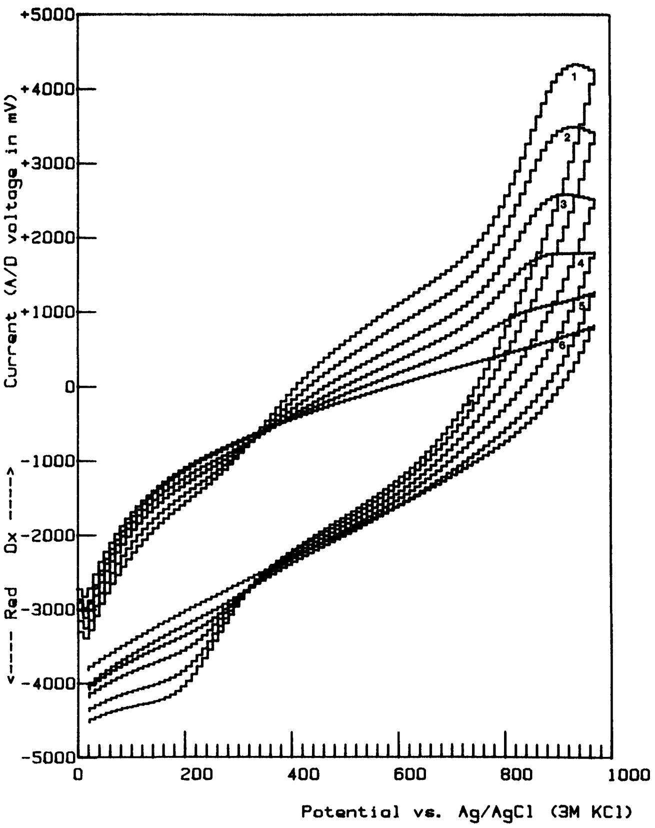

*Figure 5. Stopped-flow cyclic voltammograms of the electrolysis-product of etoposide and blank after HPLC. Scans are recorded (1) 93, (2) 105, (3) 139, (4) 163 and (5) 267 s after stopped-flow. Conditions: current range 0·5 μA F.S.; 30 μg injected; solvent methanol/0·020 M phosphate buffer pH = 8 (40 : 60 w/w); scan speed 200 mV/s; coulometric potential +500 mV versus $H^+/H_2$ couple; scan range 0 to +1000 mV versus the Ag/AgCl (3 M KCl) electrode.*

ance of the same experiment, without on-line electrolysis, the limiting currents of etoposide remain constant, precluding any influence from diffusion or adsorption.

The data from the voltammograms are used to calculate the half-life of the radical. At room temperature, in methanol/0·020 M phosphate buffer pH = 8 (40:60 w/w), the half-life is 100 s (n = 3 s.d. = 1 s).

### Conclusions

Application of a post-column ESA Coulochem 5100A coulometric module, in combination with computerized cyclic voltammetric detection enables generation, detection and identification of the free radical of etoposide.

Using a methanol-phosphate buffer (pH = 8) mixture (40:60 w/w) as mobile phase, the forward scan of the on-line cyclic voltammogram of etoposide shows two oxidation steps, caused by double one-electron transfer.

Post-column electrolysing at +500 mV versus the $H^+/H_2$ couple (pH = 8), results in the total disappearance of the first oxidation step, whereas a reduction wave arises: the limiting currents of this wave and the second oxidation step are of equal height, indicating one-electron processes.

The half-life time of the free radical proved to be dependent on experimental conditions [15]. In the system described here, the half-life time of the oxidation-product, the free radical, using the stopped-flow method, is 100 s.

Decreasing the pH to pH = 2·2 leads to merging of the two oxidation steps to a single two-electron transfer, disabling radical formation.

The half-life time and yield of the coulometrically generated free-radical of etoposide under physiological conditions (pH 7–8) are sufficient to assume involvement in enzymatic reactions *in vivo.* Resulting species are believed to be involved in activation mechanisms, as well as in toxicity-enhancing mechanisms. It is of importance to determine if, and in which way, the free radical is involved in antineoplastic action.

### References

1. Cavalli, F., Sonntag, R. W., Jungi, F., Sennand, H. J. and Brunner, K. W., *Cancer Treatment Reports,* **62** (1978), 473.
2. Issell, B. F. and Crooke, S. T., *Cancer Treatment Reviews,* **6** (1979), 107.
3. Rivera, G., Avery, T. and Pratt, C., *Cancer Chemotherapy Reports,* **59** (1975), 743.
4. Pavone-Macaluso, M., *European Urology,* **2** (1976), 138.
5. Loike, J. D. and Horwitz, S. B., *Biochemistry,* **15** (1976), 5443.
6. Horwitz, S. B. and Loike, J. D., *Lloydia,* **40** (1977), 82.
7. Kalwinsky, D., Sinkule, J. and Fridland, A., *Proceedings of the American Association for Cancer Research,* **23** (1982), 198.
8. Estape, J., Milla, A., Agusti, A., Lloret, J. S., Palacin, A. and Soriano, E., *Cancer N.Y.,* **43** (1979), 72.
9. Sinha, B. K., Trush, M. A. and Kalyanaraman, B., *Proceedings of the American Association for Cancer Research,* 25 (1984), 354.
10. Sinha, B. K., Trush, M. A. and Kalyanaraman, B., *Biochemical Pharmacology,* **34** (1985), 2036.
11. Sinha, B. K., Trush, M. A. and Kalyanaraman, B., *Biochemical Pharmacology,* **32** (1983), 3495.
12. Van Maanen, J. M. S., Holthuis, J. J. M., Gobas, F., de Vries, J., van Oort, W. J., Emmelot, P. and Pinedo, H. M., *Proceedings of the American Association for Cancer Research,* **24** (1983), 319.
13. Wozniak, A. J., Hande, K. R. and Ross, W. E., *Proceedings of the American Association for Cancer Research,* **24** (1983), 319.
14. Sinha, B. K. and Myers, C.E., *Biochemical Pharmacology,* **33** (1984), 3725.
15. Van Maanen, J. M. S., de Ruiter, C., Kootstra, P. R., de Vries, J. and Pinedo, H. M., *Free Radical Research Communications,* **1** (1986), 263.
16. Ploegmakers, H. H. J. L., Mertens, M. J. M. and van Oort, W. J., *Analytica Chimica Acta,* **174** (1985), 71.
17. Holthuis, J. J. M., van Oort, W. J., Romkens, F. M. G. M., Renema, J. and Zuman, P., *Journal of Electroanalytical Chemistry,* **184** (1985), 317.
18. Bond, A. M., *Modern Polarographic Methods in Analytical Chemistry* (Marcel Dekker, New York, 1980), p. 227.
19. *The Model 5100A Coulochem Detector Instruction Manual* (ESA Inc., March 1985), 7–30.
20. Evans, D. H., Jimenez, P. J. and Kelly, M., *Journal of Electroanalytical Chemistry,* **163** (1984), 145.

